# Tablet PC use directly affects children’s perception and attention

**DOI:** 10.1038/s41598-021-00551-9

**Published:** 2021-10-27

**Authors:** Nicole Wetzel, Dunja Kunke, Andreas Widmann

**Affiliations:** 1 Leibniz Institute for Neurobiology, Brenneckestr. 6, 39119 Magdeburg, Germany; 2 Center for Behavioral Brain Sciences, Magdeburg, Germany; 3 University of Applied Sciences Magdeburg-Stendal, Magdeburg, Germany; 4 Institute of Psychology, Leipzig University, Leipzig, Germany

**Keywords:** Psychology, Attention, Perception, Neuroscience, Cognitive neuroscience

## Abstract

Children currently grow up with a marked increase in interactive digital mobile media. To what extent digital media directly modulate children’s perception and attention is largely unknown. We investigated the processing of task-irrelevant auditory information while 37 children aged 6;8–9;1-years played the identical card game on a tablet PC or with the experimenter in reality. The sound sequence included repeated standard sounds and occasionally novel sounds. Event-related potentials in the EEG, that reflect sound-related processes of perception and attention, were measured. Sounds evoked increased amplitudes of the ERP components P1, P2 and P3a during the interaction with the tablet PC compared to the human interaction. This indicates enhanced early processing of task-irrelevant information and increased allocation of attention to sounds throughout the interaction with a tablet PC compared to a human partner. Results suggest direct effects of typical situations, where children interact with a tablet PC, on neuronal mechanisms that drive perception and attention in the developing brain. More research into this phenomena is required to make specific suggestions for developing digital interactive learning programs.

## Introduction

Children currently grow up with increased exposure to digital media use^1^, in particular access to mobile media devices^2^. Several studies confirmed that well-designed digital interactive learning programs can support the enhancement of cognitive skills and knowledge^3^. Current discussions on digital media use as a means to enhance teaching and to improve learning success are common. Digital media use can have beneficial, but also harmful effects on cognitive functions and behavior^4^. It is therefore important to understand how digital media use influences cognitive functions in the developing brain. Particularly, immediate effects of digital media use on perception and attention potentially modulate further information processing and hence learning and memory. The interaction of digital media use with attention is even more important from a developmental perspective, because attention develops significantly throughout childhood^5–7^. In this study, we used an ecologically highly valid situation and compared perception and attention of task-irrelevant auditory information when children played alternately with a tablet PC or a human opponent.

**Figure 1:**
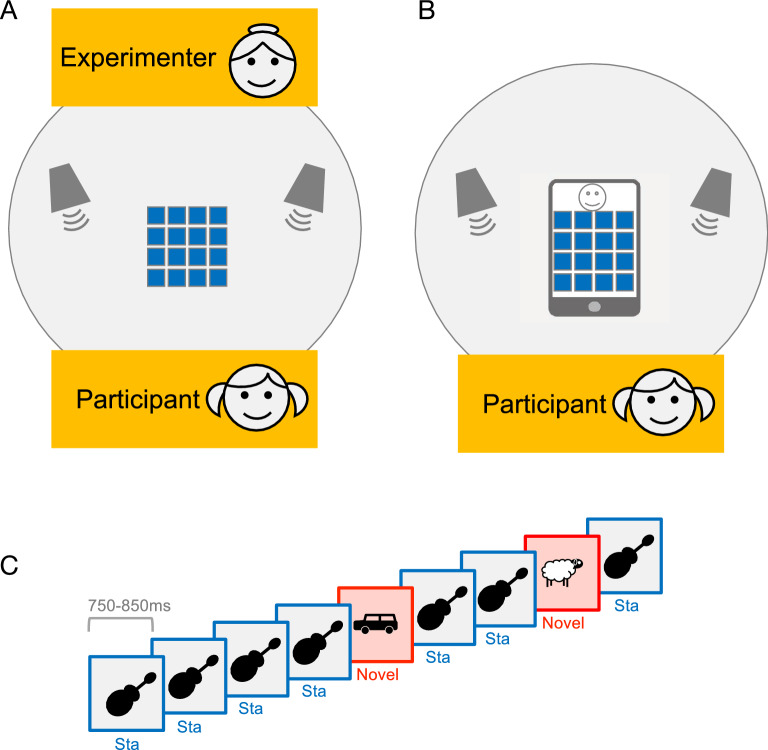
Setting and oddball paradigm. Children sat at a table and played the game *Memory* alternately with an experimenter sitting face to face (**A**) or with a *Memory* game App on a Tablet PC (**B**). In both conditions, a sound sequence consisting of repetitive standard sounds (sound of guitar) and rarely and randomly interspersed new environmental sounds was presented during the game (**C**). Children were instructed to ignore the sounds. The figure was created using Microsoft Powerpoint (version 16.52, https://www.microsoft.com/de-de/microsoft-365/microsoft-office).

Selective attention, the ability to focus voluntarily on relevant information and to ignore irrelevant information, is required when interacting with digital media. Voluntary attention and involuntary distraction of attention can be experimentally investigated using versions of the oddball paradigm, that include a sequence of repeated standard sounds and rarely and randomly presented novel sounds while participants perform a task not related to the sounds^8^. Even if the sound sequence is not relevant for the task, sounds are processed up to a certain level (e.g. sounds are encoded on the level of the auditory cortex^9^) and sequential regularities as for example repeated standards are assumed to generate the basis for an internal model predicting the auditory environment^10^. The occurrence of an unexpected sound outside the current focus of attention, that violates the prediction, can capture attention and trigger evaluation processes allowing the adaptation of behavior or mental processes. These orienting and evaluation processes may include capacity limited processes resulting in impaired performance in a task at hand^8^. Different stages of sound processing and attentional orienting and evaluation have been associated with different components in the event-related potentials (ERPs) in the electroencephalogram (EEG). The EEG is a noninvasive and time sensitive method to measure spontaneous as well as stimulus-related neuronal electrical brain activity on the surface of the scull. We applied EEG to measure brain activity involved in perception and attention in response to a task-irrelevant oddball sound sequence while children played a card game with a human or on a tablet PC (Fig. 1). In children, sounds frequently evoke prominent P1 and P2 components in the ERP that are associated with different steps of information processing such as stimulus encoding processes (P1)^9^ and early classification and attention processes (P2)^11^. In addition, we measured the ERP component P3a that reflects the differential processing of novel and standard sounds and is associated with the orienting of attention to an unexpected sound^8^. In the age range of the present study (6;8–9;1-years), these ERP components can be observed reliably, when applying an oddball sound sequence, even if amplitudes, latencies, or topographies change throughout development^12–14^.

In the present study, we applied an ecologically highly valid situation. Children played the card game *Memory* either on a tablet PC with a virtual opponent or in reality with a human opponent, while they were asked to ignore irrelevant sounds. We expected that sounds would be processed despite not being relevant for playing the card game (reflected by ERP components P1 and P2)^14^. Unexpected and task-irrelevant novel sounds were expected to involuntarily capture attention (reflected by ERP component P3a)^15^. The impact of playing on the tablet PC versus playing with a human opponent on attention was an open question due to the lack of previous applicable findings.

## Materials and methods

### Participants

A total of 37 children aged 6;8–9;1 years;month (mean age of 8 years, range of 2;6 years;months, 17 female, 36 right-handed) participated in the study. Ethical approval was obtained from the local ethics committee (Medical Faculty of the University of Magdeburg). Families of 6 local schools expressed an interest to participate in this study and were subsequently invited, after the schools agreed to hand out details of our research project to parents. The sample included only German-speaking participants. A total of 26 children participating in the study used computers for gaming at least once a week and 11 children less often than that. Children and parents gave oral and written informed consent. Parents denied any medication affecting the central nervous system, neurological disorders, hearing disorders, or attention deficit hyperactivity disorder. Participation in the study was rewarded by vouchers for a local toy shop.

### Task

Participants were asked to play the game *Memory* either with a human opponent in reality or with a virtual opponent on a tablet PC and to ignore a task-irrelevant sound sequence. The game in the tablet PC condition was presented with the App *Fiete Match.* The virtual opponent is an animated Sailor who shows different emotional reactions (e.g. joy, anger, boredom) while playing the game. The participants played with 16 cards displaying marine animals in both conditions. The game contains pairs of identical cards that are shuffled and placed, face down in a block of 4 × 4 cards. Each player has to select two cards and place them face up in the 4 × 4 array for all players to see what is on the cards. If the selected cards are not a matching pair—they are replaced, face down in the same position. If it is a matching pair, the respective player wins the pair of cards. The goal for each player is to win as many card pairs as possible and this can be done best, by memorizing the position of all the previously shown cards. In the human partner condition, the participants played with real cards that were identical regarding size, pictures (identical marine animals), colors and arrangements to the cards on the tablet PC. The human opponent was instructed to match the difficulty of the game to the difficulty level of the App, that was constantly set to the lowest level (*easy*), and to allow a similar percentage of winnings for the participant.

### Sounds

Sounds were identifiable environmental sounds, for example the noises of farm animals like a duck quacking, a pig grunting or a person sneezing, etc. (database described by^16^). The standard sound was the sound of a guitar. Sounds had a duration of 200 ms including a raised cosine windowed fade-in and fade-out of 10 ms each. Sounds were presented with an average loudness of 61.4 dB SPL as measured by a sound pressure meter, Phonic PAA3 from Phonic (Taiwan). Loudness of sounds was equalized using root mean square amplitude.

### Apparatus and experimental situation

The experiment was performed in an acoustically attenuated and electromagnetically shielded booth. In the human partner condition, the participant and the young adult sat opposite each other at a round table with the cards placed on a white surface directly in front of the participant. The distance between eyes and cards was approximately 40 cm. Two speakers were placed, 68 cm apart on the table facing the participant. In the tablet PC condition, the tablet was placed directly in front of the participant. The auditory stimuli were presented via loudspeakers (Bose Companion 2 series III Multimedia speaker system) located on the left and the right, 60 cm from the child respectively. The experimental stimulation was presented via Psychtoolbox (Version 3.0.15)^17^ using Octave (Linux, Version 4.0.0). The *Memory* game was presented using the App Fiete Match Version 1.3.0, Ahoiii Entertainment UG (Germany) on an Apple iPad, iOS version 5.1.1, Apple (USA). For the experiment the App Fiete Match was set on marine animals with 16 cards.

### Procedure

Every child was introduced to the procedure and asked if he or she wanted to perform the task. The experimenter answered any questions. Once the child agreed to the EEG procedure, the appropriate size EEG cap was placed and prepared. The child sat in the soundproof booth with the EEG cap on and the *Memory* game was explained and shown including the scoring system for both the tablet PC and the human partner condition. During the human partner condition, the experimenter sat opposite the child in the booth whereas in the tablet PC condition, the child was alone in the booth with the tablet. The experimenter could see and hear the child via a camera for safety. The experimenter and the children did not chat during the game. This was agreed on with the children before the game started and both the children and the experimenter stuck to it. The party with the most cards at the end of the game won and scored a point. This applied to both the virtual and the human partner condition. In the human partner condition, student assistants were instructed to allow for the same individual percentage of wins as the App does. The children won 62% of the games in the human partner condition and 59% of the games in the tablet PC condition. There was no statistically significant difference in the win rate of the children between conditions as indicated by moderate evidence for the null hypothesis (BF_10_ = 0.33; *p* = 0.25). The experimenter documented her observation of whether the child made an effort to win. This was observed for 35 of 37 children during the entire experiment. Two children showed a decline in motivation at the very end of the experiment. Furthermore, we asked children whether they had fun playing the card game. This was affirmed by all children. One student assistant participated as the human opponent for 19 of the children and the other assistant for 18 children. Both student assistants (female) were experienced in working with children. The experimenter responded with what would be considered appropriate emotional reactions in a card game, that is joy when winning and mild disappointment when losing. The digital opponent showed similar emotional reactions in the same situations.

There were two blocks for each condition (human partner and tablet PC), thus a total of 4 blocks. The order of blocks was randomized. A total of 480 novel sounds (20%) and 1920 standard sounds (ca. 80%) were presented, that is, each block comprised 120 novel sounds and 480 standard sounds. The order of sounds was pseudo-randomized separately for each participant and block with the restriction that at least two standard sounds were presented before a novel sound. Each novel sound was presented once per block. In the whole experiment, each novel sound was presented four times. Sounds were presented with a variable stimulus onset asynchrony of 750, 775, 800, 825 and 850 ms that occurred with equal probability. The sound sequence started when the game started, stopped when the game finished and continued when the next game started. This approach prevents the contamination of sound-related brain activity caused by mixing and distributing cards (human partner condition) or by restarting the game (tablet PC condition). If the sound sequence continued with a novel sound within the first two trials, automatically one or two standard sounds were prepended. This resulted in a slightly different number of presented trials for every child. The actual probability of the presentation of a novel sound varied between 19.8% and 20%.

The session lasted 90 min including breaks and EEG preparation. If parents agreed, sweeties and soft drinks were offered in the break time between the blocks.

### EEG and data analyses

#### EEG recording

The electroencephalogram (EEG) was recorded via an ActiChamp amplifier with a 31 active electrode Braincap (Brain Products GmbH, Gilching, Germany). The sampling rate was 500 Hz. The following electrodes were placed according to the extended 10–20 system: Fp1, Fp2, F7, F3, Fz, F4, F8, FC5, FC1, FC2, FC6, C3, Cz, C4, CP5, CP1, CP2, CP6, P7, P3, Pz, P4, P8, O1, O2, Oz, and at the left (M1) and right (M2) mastoids. Three electrodes recording the horizontal and vertical electrooculogram (EOG) were positioned to the left and right of the outer canthi of the eyes and below the left eye. The reference electrode was placed at the tip of the nose.

#### EEG preprocessing

EEG data analysis was performed with MATLAB software (version R2020a, https://de.mathworks.com/products/matlab.html) and the EEGLAB toolbox^18^. Data were filtered offline with a 0.1 Hz high-pass filter (Hamming windowed sinc FIR filter, order = 8250, transition band width = 0.2 Hz)^19,20^ and a 48 Hz low-pass filter (Hamming windowed sinc FIR filter, order = 414, transition band width = 4 Hz). The data were segmented into epochs of 0.8 s duration including a 0.2 s pre-stimulus baseline. Bad channels with a robust z-score of the robust standard deviation larger than 3 (a single channel in 2 of the participants)^21^ and epochs with amplitude differences exceeding 1.5 mV at any channel were removed from the data. Data were corrected for artifacts using independent component analysis. To improve the decomposition, ICA was computed on the epoched, but not baseline corrected raw data^22^, excluding bad channels and epochs, filtered by a 1 Hz high-pass filter (continuous data; Hamming windowed sinc FIR filter, order = 1650, transition band width = 1 Hz) and a 48 Hz low-pass filter (see above) using AMICA^23^. The obtained demixing matrix was then applied to the 0.1–48 Hz filtered data^24^.

Eye movement related artifact ICs were classified with the ICLabel EEGLAB plugin^25^ and re-checked manually. Four to six eye movement related ICs were detected in all subjects (*M* = 5, *Mdn* = 5, *SD* = 0.33; blinks/eyelid induced artifacts, horizontal and vertical movements of the corneo-retinal dipole, spike potentials related to horizontal and vertical eye movements). Artifact ICs reflecting activity of frontal, temporal, and occipital muscles (note that participants were actively moving in both conditions) were classified manually informed by topography, power spectrum and evoked activity in the average across trials. Only components showing a topography specific for one or more muscles were considered as muscle activity ICs. Components showing any indication of neural activity, for example alpha activity, a 1/f-like power spectrum, or sound onset locked activity in the average across trials were never considered as artifact (eye movement or muscle activity) ICs. Three to ten muscle activity related ICs were detected per subject (*M* = 6.7, *Mdn* = 7, *SD* = 2.6). Eye movement and muscle IC activity was subtracted from the data.

Trials were baseline corrected. Bad channels were interpolated using spherical spline interpolation. Trials with amplitude differences exceeding 200 µV, the first two trials per block, and two standard trial following a novel trial were excluded from the analysis. Grand-average waveforms were computed from the individual average ERPs for each condition and stimulus type. Descriptive statistics on the number of included trials per condition are shown in Table 1.

**Table 1:** Descriptive statistics on the number of included trials per condition.

	Human partner		Tablet PC	
	Standard	Novel	Standard	Novel
Mean	449.1	228.8	448.0	228.9
Median	459	234	459	235
SD	37.5	18.2	41.0	21.2
Min	294	147	231	118
Max	473	240	476	240

#### PCA analysis

As traditional ERP analyses suffer from the relatively arbitrary definition of analysis time windows and the overlap of ERP components, we performed a temporal principal component analysis with the ERP PCA Toolkit MATLAB toolbox by Dien^26^. PCA is particularly recommended for developmental populations reducing problems due to the enhanced noise level^27^. The PCA identifies the constituent components of the ERP and provides dependent measures of these components for inferential testing. PCA is based on factor-analytic procedures using eigenvalue decomposition to extract linear combinations of variables (latent factors) accounting for patterns of covariance observed in the EEG data, presumably due to ERP components^28^. The PCA was computed on the individual average ERP responses. PCA was computed using Promax rotation (κ = 3) with a covariance relationship matrix and Kaiser weighting (in a strict sense exploratory factor analysis rather than PCA was performed; however, as the term PCA is most commonly used in the field we also use this terminology here). According to Horn’s parallel test (as implemented in the R psych package)^29^ nine components were retained explaining 96.3% of the variance. Components were identified relying on their characteristic latencies and scalp distribution. Components are ordered by explained variance and not chronologically by peak latency. P1 was reflected in component 3 (peak latency 112 ms). P2 was reflected in component 5 (peak latency 170 ms). Early P3a was reflected in component 2 (peak latency 286 ms). Late P3a was reflected in component 4 (peak latency 364 ms).

#### Statistical analysis

Effect size estimates and 95% confidence intervals are reported for all effects and PCA component scores (in square brackets) as recommended by Cumming^30^. ERP PCA scores (Tables 2 and 3) were tested with Bayesian repeated measures ANOVAs computed separately for each PCA component including the factors *condition* (human partner vs. tablet PC) and *stimulus type* (standard vs. novel) at the electrode where the component peak was expected based on our knowledge of the children ERP literature (Fz for P1, Cz for P2, Fz for early and late P3a). Bayes factors (*BF*_10_) were estimated in R using 50,000 Monte-Carlo sampling iterations and scaling factors r = 0.5 for fixed effects (corresponding to the default “medium” effect size prior for fixed effects in the R Bayes-Factor package)^31^ and r = 1 for the participant random effect (corresponding to the default “nuisance” prior for random effects in the R Bayes-Factor package). We compared all models (constrained by the principle of marginality) with the null model (*BF*_10_) and additionally evaluated main effects and interactions by comparing the models containing a main effect or interaction to the equivalent models stripped of the effect excluding higher order interactions (“Baws Factor” or “Inclusion Bayes Factor based on matched models”`, reported as BFIncl)^32^. Following Lee and Wagenmakers^33^, data were interpreted as moderate evidence in favor of the alternative hypothesis if BF_10_ was larger than 3 or strong evidence if *BF*_10_ was larger than 10. Data were interpreted as moderate evidence in favor of the null hypotheses if *BF*_10_ lower than 0.33 or as strong evidence if *BF*_10_ was lower than 0.1. *BF*_10_ between 0.33 and 3 were considered as anecdotal (we termed this weak) evidence.

**Table 2:** Mean component scores of the PCA components.

	Human partner		Tablet PC	
	Standard [95% CI]	Novel [95% CI]	Standard [95% CI]	Novel [95% CI]
P1	1.38 [ 1.20 1.56]	1.32 [ 1.09 1.55]	1.60 [ 1.35 1.84]	1.54 [ 1.28 1.81]
P2	0.94 [ 0.72 1.17]	1.54 [ 1.16 1.93]	1.54 [ 1.26 1.83]	2.03 [ 1.63 2.43]
earlyP3a	− 1.47 [− 1.79 − 1.16]	0.70 [ 0.37 1.03]	− 1.39 [− 1.67 − 1.11]	0.83 [ 0.50 1.15]
lateP3a	− 1.11 [− 1.37 − 0.85]	− 0.58 [− 0.94 − 0.22]	− 1.22 [− 1.52 − 0.92]	− 0.17 [− 0.55 0.21]

**Table 3:** Novel minus standard difference mean component scores of the PCA components including [95% CI], Cohen’s *d* effect size, and Bayesian *t* test *BF*_10_.

	Human partner			Tablet PC		
	Nov. minus Sta	*d*	*BF*_10_	Nov. minus Sta	*d*	*BF*_10_
P1	− 0.06 [− 0.23 0.10]	− 0.13	0.235	− 0.05 [− 0.20 0.10]	− 0.12	0.225
P2	**0.60 [ 0.25 0.96]**	**0.56**	**21.585**	**0.48 [ 0.13 0.84]**	**0.46**	**4.726**
earlyP3a	**2.17 [ 1.77 2.57]**	**1.79**	**1.4 × 10**^**10**^	**2.21 [ 1.84 2.58]**	**1.99**	**2.5 × 10**^**11**^
lateP3a	**0.53 [ 0.20 0.87]**	**0.53**	**12.555**	**1.05 [ 0.72 1.38]**	**1.06**	**8.2 × 10**^**4**^

Relevant results are marked in bold.

Bayesian analyses were complemented by frequentist statistics using the identical repeated measure ANOVA designs. An alpha-level of 0.05 was defined for all statistical tests. Statistically significant results were reported including the η^2^ effect size measure. Follow-up *t*-tests (two-sided) were computed for statistically significant interactions.

All methods were carried out in accordance with relevant guidelines and regulations.

## Results

**Figure 2:**
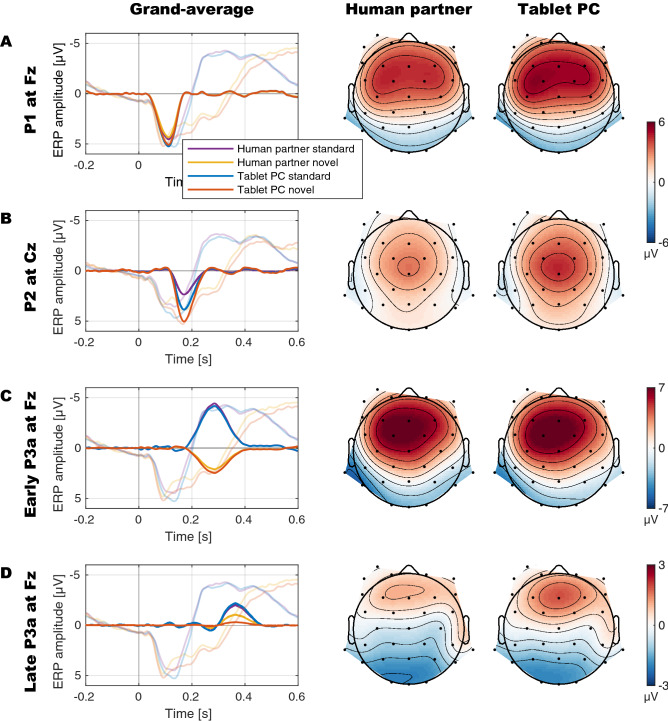
Grand-averages (transparent lines) and component loadings reflecting component time courses (opaque lines) and component topographies of the analyzed PCA components scaled to µV (that is, the portion of the recorded waveform accounted for by each component); (**A**): P1; (**B**): P2; (**C**): early P3a; (**D**): late P3a. The component waveforms are computed as product of component loading times component score times *SD* per condition averaged across participants (for a proof see Appendix of Dien)^34^. For P1 and P2 components (panels **A** and **B**) the topographies reflect the average of standard and novel trials per condition to illustrate the human partner vs. tablet PC *condition* main effect. For early and late P3a components, typically defined as difference components (panels **C** to **D**), the topographies reflect the difference of novel minus standard trials to illustrate the *condition* by *stimulus type* interaction effect. P1 (panel **A**) and P2 (panel **B**) components were significantly decreased in the human compared to the tablet PC condition (P2 was also enhanced in response to novel compared to standards; note that the orange waveform representing the tablet PC novel—overlaps with the blue waveform representing the human standard in panel B). The early P3a (in the novel minus standard difference waveform) was observed in both conditions, but its amplitude was not modulated by condition (**C**). The late P3a was also observed in both conditions and was decreased in the human condition compared to the tablet PC condition (**D**).

Main results revealed larger sound-related mean amplitudes of P1 and P2 components when children interacted with a virtual partner on the tablet PC than with a human partner in reality. Significantly different amplitudes in novel and standard sound trials were observed for the P2 and both P3a components. This difference was increased for the late P3a in the tablet PC condition. The mean grand averages and the analyzed PCA components and their topographies are displayed in Fig. 2. The PCA loadings and PCA scores per condition are reported in Fig. 2 and Tables 2 and 3, respectively.

### P1

The Bayesian ANOVA favored the model including the *condition* main effect only (*BF*_10_ = 89.0). The observed P1 amplitude was smaller in the human partner compared to the tablet PC condition (main effect *condition*: − 0.22 95% CI [− 0.35 − 0.09], *BF*_Incl_ = 90.5; *F*(1,36) = 11.05, *p* = 0.002, η^2^ = 0.235). The data provide moderate evidence against an effect of *stimulus type* on P1 (− 0.06 [− 0.18 0.07]; *BF*_Incl_ = 0.281; *F*(1,36) = 0.85, *p* = 0.361, η^2^ = 0.023) and against an interaction effect of *condition* and *stimulus type* (− 0.01 [− 0.19 0.17]; *BF*_Incl_ = 0.230; *F*(1,36) = 0.01, *p* = 0.906, η^2^ < 0.001).

### P2

The Bayesian ANOVA favored the model including both *condition* and *stimulus type* main effects (*BF*_10_ = 3.6 × 10^5^). The observed P2 amplitude was smaller in the human partner compared to the tablet PC condition (main effect *condition*: − 0.54 [− 0.78 − 0.31]; *BF*_Incl_ = 1.1 × 10^3^; *F*(1,36) = 22.26, *p* < 0.001, η^2^ = 0.382) and larger in response to novel compared to standard sounds (main effect *stimulus type*: 0.54 [0.22 0.87]; *BF*_Incl_ = 1.1 × 10^3^; *F*(1,36) = 11.526, *p* = 0.002, η^2^ = 0.243). The data provide moderate evidence against an interaction effect of *condition* and *stimulus type* (0.12 [− 0.17 0.40]; *BF*_Incl_ = 0.257; *F*(1,36) = 0.692, *p* = 0.411, η^2^ = 0.019).

### Early P3a

The Bayesian ANOVA favored the model including the *stimulus type* main effect only (*BF*_10_ = 5.4 × 10^33^). The observed early P3a amplitude was more positive in response to novel compared to standard sounds (main effect *stimulus type*: 2.19 [1.81 2.57]; *BF*_Incl_ = 5.6 × 10^33^; *F*(1,36) = 138.887, *p* < 0.001, η^2^ = 0.794). The data provide moderate evidence against an effect of *condition* on early P3a (− 0.10 [− 0.26 0.05]; *BF*_Incl_ = 0. 246; *F*(1,36) = 1.907, *p* = 0.176, η^2^ = 0.050) and against an interaction effect of *condition* and *stimulus type* (− 0.04 [− 0.22 0.13]; *BF*_Incl_ = 0.246; *F*(1,36) = 0.251, *p* = 0.620, η^2^ = 0.007).

### Late P3a

The Bayesian ANOVA favored the model including both *condition* and *stimulus type* main effects and the interaction effect of *condition* and *stimulus type* (*BF*_10_ = 5.0 × 10^7^). Late P3a was observed in both conditions (main effect *stimulus type*: 0.79 [0.51 1.07]; *BF*_Incl_ = 5.2 × 10^7^; *F*(1,36) = 33.244, *p* < 0.001, η^2^ = 0.480) but was reduced in the human partner compared to the tablet PC condition (interaction effect of *condition* and *stimulus type*: − 0.52 [− 0.89 − 0.15]; *BF*_Incl_ = 2.608; *F*(1,36) = 8.114, *p* = 0.007, η^2^ = 0.184; human partner: *t*(36) =  − 3.207, *p* = 0.003; *BF*_10_ = 12.555; tablet PC: *t*(36) =  − 6.425, *p* < 0.001; *BF*_10_ = 8.2 × 10^4^). The data provide weak evidence against an effect of *condition* on the late P3a (main effect *condition*: − 0.15 [− 0.34 0.04]; *BF*_Incl_ = 0.392; *F*(1,36) = 2.492, *p* < 0.123, η^2^ = 0.006; that is, the *condition* main effect is included in the preferred model due to the principle of marginality).

## Discussion

Playing a card game with a virtual opponent on the tablet PC has immediate effects on the processing of task-irrelevant auditory information, reflected by increased amplitudes of the ERP components P1 and P2 and on attentional orienting and evaluation, reflected by increased amplitude of the ERP component P3a, compared to a condition where 6;8–9;1-year-old children played exactly the same card game with a human opponent in reality.

The P1 ERP component of the EEG peaks around 100 ms after sound onset over frontal brain areas. This component reflects early cortical processing of sounds presumably on the level of the auditory cortex^9^. Results demonstrate increased activity on the level of the auditory cortex in response to task-irrelevant sounds when children play with a tablet PC compared to playing the same game with a human partner. The lack of differences between standard and novel sounds at this stage of information processing indicates enhanced processing of all sound types independent from their novelty. Furthermore, also the amplitudes of the following sound-related P2 component are enhanced during tablet PC use. The P2 component reflects activity of different sources and at least partly the auditory driven output of the mesencephalic reticular activating system^12,35^. The functional role of P2 underlying processes is not yet well understood. It has been proposed that the P2 reflects processes of stimulus classification as well as attention^36^. Significant events such as target stimuli, but also new or emotional stimuli can cause increased P2 amplitudes^37,38^, even in children^39^. In contrast to early encoding reflected by the P1 component, P2 amplitudes were additionally increased in response to novel sounds compared to standard sounds indicating enhanced processing of unexpected new, but task-irrelevant sounds around 200 ms after stimulus onset. Results indicate enhanced processing of irrelevant sounds that include attention-related mechanisms during playing a card game when children interact with a tablet PC compared to interacting with a human partner.

The P1 and P2 underlying processes constitute the basis for further information processing on higher cortical levels. Different brain responses to novel and standard sounds demonstrate that the to-be-ignored sounds were processed to such an extent that the novelty of sounds captured children’s attention. This can be best observed in the difference wave (novel minus standard ERP) on the P3a component^40^. Two subcomponents of the P3a were observed reflecting differential processing of standard and novel sounds. Both the early P3a and the late P3a are proposed to reflect attention-related processes^8,41^. Note, that these components are sometimes also labeled as P3a and novelty P3^42,43^. Different cerebral sources and different sensitivity to experimental manipulations indicate that the early and late P3a do not reflect the same mechanisms^40,44^. It has been demonstrated that the late P3a reflects the orienting of attention towards unexpected task-irrelevant events as well as evaluation processes^40,45–47^. The early P3a component has been recently linked to the unspecific burst of arousal evoked by unexpected and salient distracting sounds^44^. The amplitudes of the late P3a component, peaking at 364 ms over frontal brain regions, were larger when children interacted with the tablet PC than with the human partner. Following the interpretations in the literature, the larger late P3a amplitudes indicate that children allocate more attention to task-irrelevant novel information in the tablet PC condition, compared to the human partner condition.

We conclude from our findings that children process and attend task-irrelevant auditory information to a larger extent in the tablet PC than in the human partner condition. This could be explained by different models. On the one hand the focus of selective attention might be more narrow or wide for different conditions, without sounds actually distracting attention in the sense of impaired performance in the task at hand. On the other hand, the processing of irrelevant sounds might require resources as evidenced by impaired performance in oddball tasks observed in children^5^ and adults^40^. Due to the limited information processing capacity^48^ and limited attentional resources^49^, increased processing of task-irrelevant information might indicate that less resources were allocated to the task in the tablet PC condition. We did not directly measure the allocation of attention to the task, therefore beyond the enhanced sound processing, we speculate that children allocated more resources to task-related processes or the task-related context when they interact with a human being compared to the tablet PC. This hypothesis is supported by ERP studies demonstrating that oddball sound related ERPs are a reliable marker of workload in a primary task, in particular when sounds were ignored^50^. Increased effects of decreased cognitive load on sound processing were reported by Miller et al.^51^. The level of a computer game influenced amplitudes of ERP-components reflecting early sensory processing and stimulus evaluation, that is, a more difficult level of the gaming task was accompanied by attenuated amplitudes of sound-related ERP components N1, P2 and P3 in adults^51^. The authors interpreted the N1 and P2 attenuations as indicators of reduced attention allocation to task-irrelevant sounds under high cognitive load conditions. In line with this, some studies with adults reported decreased amplitudes of the P3a component, associated to orienting of attention, when working memory load was high^52–54^, but see^55,56^. Our hypothesis on the impact of available resources is particularly in line with finding by SanMiguel and colleagues^57^. They manipulated the working memory load in the visual modality while a to-ignore oddball sound sequence, including oddball novel sounds was presented. They reported reduced amplitudes of the novel-related late part of the P3a when the working memory load was high. Even if these findings support our hypothesis that the amount of cognitive resources contingent to the human partner vs. tablet PC condition influences the extent to which irrelevant information is attended and processed, further studies are required to confirm this hypothesis and to disentangle potential influencing factors.

A strength of this study is the measurement of brain activity during a playful activity that is typical for children in a natural environment. The high ecological validity implies that the present study does not allow for the unequivocal disentangling of probable factors contributing to the different utilization of resources in the two conditions. There is a variety of resources consuming brain processes, that potentially differ between the conditions, for example motivational factors (different effort depending from human or virtual opponent), perceptual differences (slightly different visual scenes due to the difference in size between the human opponent and the tablet PC) or emotional processes (processing of social (facial) cues). In particular the interaction with a human opponent in a natural environment requires additional attentional resources for social perception and social evaluation processes^58^. To apply a typical gaming situation (where children usually play alone on a tablet PC) and to avoid the impression of surveillance of performance by the experimenter, the experimenter was not in the booth during the tablet PC condition. We cannot rule out that the absence vs. presence of the experimenter between conditions influenced the results. On the other hand, the presence of the experimenter in the tablet PC condition could also influence the results, for example by creating an additional social context in which the child may focus on the experimenter in addition to the virtual partner and feel more tempted to communicate wins and losses.

Increased distractibility of children during tablet PC use has been observed in developmental studies focusing on language acquisition and learning using digital media devices. These studies have primarily investigated effects of enriched ebooks on learning success in young children. Findings revealed beneficial and adverse effects on learning, depending on consistency between presented material and the way that the human information processing system functions^3^. Some studies indicate that animations not related to the story can distract children’ attention resulting in impaired learning success^3,59^.

## Conclusion

In the tablet PC condition, children’s processing of irrelevant auditory information and their capture of attention by task-irrelevant novel sounds was increased compared to the human partner condition. The results demonstrate direct and immediate effects of the tablet PC condition on neuronal mechanisms underlying perception and attention in the developing brain. Digital media are increasingly used for teaching purposes and the impact on attention allocation in children has to be considered when designing and using interactive learning applications. The present findings are particularly relevant for the use of learning applications that are enriched with background music or task-irrelevant background sounds. Even if different factors contributing to this result still have to be investigated in future studies, our results are based on conditions that are currently experienced by many children and provide a basis for future research on attention allocation during digital media use from a developmental neurocognitive perspective.

## Data Availability

The datasets generated during and/or analyzed during the current study are available from the corresponding author on reasonable request.
